# The performance of the WHO mutation catalog of *Mycobacterium tuberculosis* in the diagnosis of drug resistance to rifampicin-resistant strains in Wenzhou, China

**DOI:** 10.1128/spectrum.02481-25

**Published:** 2026-03-31

**Authors:** Guiqing He, Qingyong Zheng, Jing Wu, Jingjie Luan, Yuanfei Ji, Wenzhen Zhou, Xia Yu

**Affiliations:** 1 Department of Infectious Diseases, Wenzhou Central Hospital, Affiliated to Wenzhou Medical University 223520, Wenzhou, China; 2 Laboratory of Infectious Diseases, Wenzhou Central Hospital, Affiliated to Wenzhou Medical University 223520, Wenzhou, China; 3 National Clinical Laboratory on Tuberculosis, Beijing Key Laboratory on Drug-Resistant Tuberculosis, Beijing Chest Hospital, Capital Medical University 12517, Beijing, China; 4 School of Public Health (Shenzhen), Shenzhen Campus of Sun Yat-sen University 660329 , Shenzhen, China; Weill Cornell Medicine, New York, New York, USA

**Keywords:** *Mycobacterium tuberculosis*, resistance prediction, mutation catalog

## Abstract

WHO released the first and second editions of *Mycobacterium tuberculosis* complex (MTBC) mutations catalog associated with drug resistance in 2021 and 2023, respectively. The aim of this study was to evaluate the resistance prediction by mutation catalog for nine anti-tuberculosis drugs in rifampicin (RIF)-resistant tuberculosis (RR-TB) in Wenzhou, China. Over the period from 2020 to 2022, 301 RR-TB cases were continuously prospectively collected in Wenzhou Central Hospital. A total of 214 MTBC isolates were collected with drug susceptibility testing using the MGIT960 system and MYCOTB plates. Whole-genome sequencing (WGS) was performed for resistance prediction using mutation catalog by the WHO and freely available tools (TB-Profiler). Among 214 RR-TB isolates, drug resistance prediction using the WHO second edition mutation catalog demonstrated high sensitivity when stratified by the two predominant lineages. In Beijing lineage (L2, *n* = 171) isolates, sensitivity was 98.73% for RIF, 95.77% for isoniazid (INH), 89.29% for ethambutol (EMB), and 98.04% for moxifloxacin (MFX). Corresponding values in Euro-American lineage (L4, *n* = 43) isolates were 100.00% for RIF, 92.11% for INH, 94.44% for EMB, and 75.00% for MFX. Specificity exceeded 80% for all drugs except RIF in both lineages. Additionally, three potential resistance-associated mutations were determined (*ahpC*_−52C>T for INH, *embA*_−11C>A for EMB, *eis*_−10G>A for Kanamycin). Resistance prediction based on mutation catalog offers better patient-tailored treatment of tuberculosis. Integrating more new drug mutations from different geographic areas would improve the accuracy of resistance prediction.

**IMPORTANCE:**

Based on the catalog of mutations in *Mycobacterium tuberculosis* complex (MTBC) and their association with drug resistance published by the WHO in 2021 and 2023, this study evaluated the predictive performance of the catalogs for resistance to nine anti-tuberculosis drugs among 214 MTBC isolates from Wenzhou Central Hospital. The results demonstrated high accuracy of resistance prediction using the second edition. Three potential resistance-associated mutations were also identified. These findings not only confirm the reliability of the current catalog but may also provide valuable insights for future updates.

## INTRODUCTION

In 2023, an estimated 10.8 million new cases of tuberculosis were reported, the estimated 1.25 million deaths from tuberculosis (1). Globally in 2023, 0.4 million people were estimated to have developed multidrug-resistant tuberculosis (MDR-TB). Rapid determination of extensive resistance profiles is urgently needed for enabling prompt initiation of patient-tailored treatment regimens, which calls for universal access to drug-susceptible testing.

Although phenotypic drug susceptibility testing (pDST) takes up to 6 weeks to complete and is still the reference standard for most drugs, it is unreliable and/or not standardized for several drugs according to the WHO guidelines (2), and cannot fully guide clinical treatment (3, 4). In the last decade, genotypic drug susceptibility tests such as Xpert MTB/RIF (Xpert) for rifampicin (RIF), MTBDRplus/sl line probe assays for RIF and isoniazid (INH), and whole-genome sequencing (WGS) have relied on genotypic resistance prediction (5–7). For better predicting the clinically relevant resistance phenotypes from genetic data, the WHO issued the first edition of *Mycobacterium tuberculosis* complex (MTBC) mutations associated with drug resistance from MTBC isolates (>38,000) worldwide in 2021 (8). According to the increase in data on the clinical MTBC isolates with genotypes and phenotypes, more MTBC isolates (>52,000) were used to identify new genomic variants associated with phenotypic resistance to the new and repurposed TB drugs, and the second edition of the drug mutation catalog was issued in 2023 (9). Drug resistance variants were graded into five categories: (group 1) associated with resistance, (group 2) associated with resistance-interim, (group 3) uncertain significance, (group 4) not associated with resistance-interim, and (group 5) not associated with resistance.

Mutation sites associated with anti-tuberculosis drug resistance vary among different countries and regions, especially in high TB burden countries. China is the third-highest tuberculosis burden country, which is lower than Indonesia and India. Meanwhile, China is one of 30 countries with MDR or RIF-resistant tuberculosis (RR-TB), accounting for 7.3% of the total drug-resistant tuberculosis (1). Integrating more resistant MTBC isolates from high tuberculosis burden countries and regions would boost the development of genome-based resistance diagnosis worldwide, improving the geographical representation of data in the catalog.

In this study, we aimed to evaluate the performance of the WHO mutation catalog (first and second editions) in predicting drug resistance using 214 RR-TB strains isolated in Wenzhou, China. In addition, some potential drug-resistant mutations were identified, which were not included in the current five categories by the WHO mutation catalog.

## MATERIALS AND METHODS

### Data sources

From January 2020 to December 2022, we prospectively and continuously collected RR-TB patients who were admitted to Wenzhou Central Hospital. Additionally, patients diagnosed with RR-TB in other hospitals in the Wenzhou region were also assigned to Wenzhou Central Hospital for treatment. RIF resistance was determined by Xpert or MGIT960 using the baseline sputum, the corresponding culture-positive isolates identified as MTBC were collected as the baseline strains.

### Phenotypic data

In this study, drug susceptibility experiments for RIF, INH, streptomycin (SM), ethambutol (EMB), ofloxacin (OFX), moxifloxacin (MFX), amikacin (AMK), kanamycin (KM), and ethionamide (ETO) were carried out using MYCOTB plates (Thermo Fisher Scientific, Waltham, MA, USA), and two operators read the minimum inhibitory concentration (MIC) of each drug, and a third operator read the conflicting results. The critical concentrations for the tested drugs were as follows: 0.5 μg/mL for RIF; 0.12 μg/mL for INH; 2.0 μg/mL for EMB; 2.0 μg/mL for OFX; 0.5 μg/mL for MFX; 4.0 μg/mL for AMK; 5.0 μg/mL for KM; 2.0 μg/mL for SM; and 2.5 μg/mL for ETO (10, 11). The MICs for quality control strain H37Rv (ATCC27294) were determined using each lot of the prepared microtiter plates, and the results for the tested drugs were within the expected range (12).

### Genotypic data

This study performed comprehensive total DNA extraction work on various biological samples using advanced nano-magnetic bead technology. After nucleic acid extraction, the extracted nucleic acids were precisely quantified using a Qubit instrument to ensure the accuracy of subsequent experiments. Next, the Tn5 method was applied to construct the library, which is an efficient and widely used technique in genomics research. After constructing the library, Qubit and quantitative polymerase chain reaction were used to further ensure that the quality and concentration of the library met the sequencing requirements. After all preparations were completed, we performed high-throughput sequencing using the advanced Illumina sequencing platform to obtain high-quality sequencing data.

Sequencing data were quality checked using FastQC software, and the raw data were de-joined and de-low-quality bases processed using Trimgalore software (https://github.com/FelixKrueger/TrimGalore). The data were analyzed directly using the TB-Profiler software (version 5.0.1) (13), an analysis tool that may incorporate certain resistance-associated mutations beyond those listed in the WHO catalog. All relevant resistance mutation results were subsequently obtained. Bowtie2 software (https://bowtie-bio.sourceforge.net/bowtie2/index.shtml) was applied to align the processed high-quality sequencing data to the reference genome of H37Rv (ATCC 27294). SNPs were called by using the snippy tool (https://github.com/tseemann/snippy). SNP-fixed mutations were annotated using the WHO first and WHO second edition resistance lists, with the minimum frequency threshold of 10% and the minimum comparison quality threshold of 30. Multiple resistance genes to nine common clinical anti-tuberculosis drugs were detected by WGS in the included isolates.

### Classification of mutant genes

According to the WHO mutation catalog (9), genotypic mutation sites were categorized into five groups: (i) associated with resistance; (ii) associated with resistance-interim; (iii) uncertain significance; (iv) not associated with resistance-interim; and (v) not associated with resistance. According to the TB-Profiler, genotypic mutation sites were categorized into three groups: (i) sensitive; (ii) resistant; and (iii) indeterminate.

### Analysis of drug resistance correlation and unclassified WHO genotypic mutation sites

The nine drugs were analyzed for drug resistance correlation using GraphPad Prism (version 9.1.0), RStudio (version 2024.12.0+467), and IBM SPSS (version 27). The collected genotypic mutation sites of MTBC isolates were classified by the WHO first edition and second edition mutation catalogs and TB-Profiler definitions, and the genotypic mutation sites associated with resistance, occurring more than twice and not defined by the WHO mutation catalog, were counted for each of the nine drugs, using the WHO second edition mutation catalog as the standard. Unclassified mutations specifically detected in drug-resistant isolates (*n* ≥ 2) without group 1 or group 2 by the WHO second edition mutations were categorized as internal group 6.

### Construction of evolutionary trees

Resistance sites were filtered out using scripts, and Perl code was used to determine the pedigree of all samples by their evolved specific sites. RAxML software (version 8.2.12) was employed to construct the phylogenetic tree (14). We used the general time reversible model and gamma distribution to simulate nucleotide substitutions and variation rates at different sites, considering different gene frequencies. The model was corrected for Lewis deviation and resampled 500 times (Bootstrap = 500). Finally, a phylogenetic tree visualization website called ITOL was used (https://itol.embl.de/). The annotation of phenotypic resistance was conducted using the DATASET_COLORSTRIP, while the WHO second edition classification and TB-Profiler classification were facilitated by DATASET_BINARY. The highlighting of genotypic mutation sites that were not classified by the WHO and may be associated with resistance was achieved by employing color.

## RESULTS

**Fig 1:**
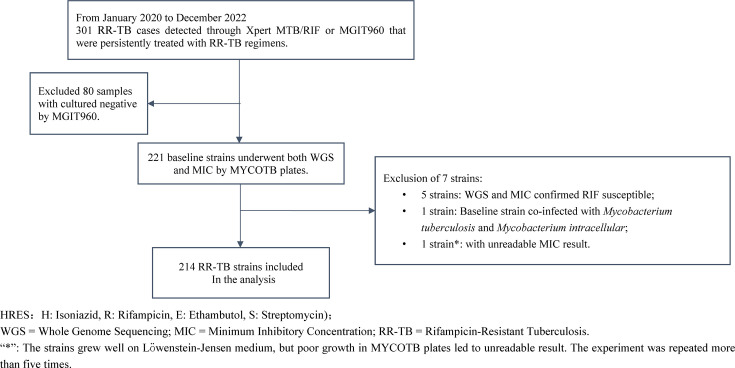
Flowchart of study inclusion and exclusion.

A total of 301 RR-TB patients were included from the RR-TB treatment cohort at Wenzhou Central Hospital in Wenzhou, China, between January 2020 and December 2022, and RIF resistance was determined by Xpert or MGIT960 using the baseline sputum. Among these, 80 cases were excluded for culture-negative by MGIT960, and the remaining 221 baseline strains were included for performing WGS and determination of MIC. Seven MTBC strains were excluded (one mixed infection with TB and *Mycobacterium intracellular*, five strains with RIF-susceptible results by WGS and MIC, and one strain with an unreadable MIC result). Finally, a total of 214 RR-TB strains were included in the analysis (Fig. 1). Of note, 199 strains were confirmed as RIF-resistant by MYCOTB plates, reflecting minor discrepancies between MGIT960 and MYCOTB results due to methodological differences and resistance criteria.

**Fig 2:**
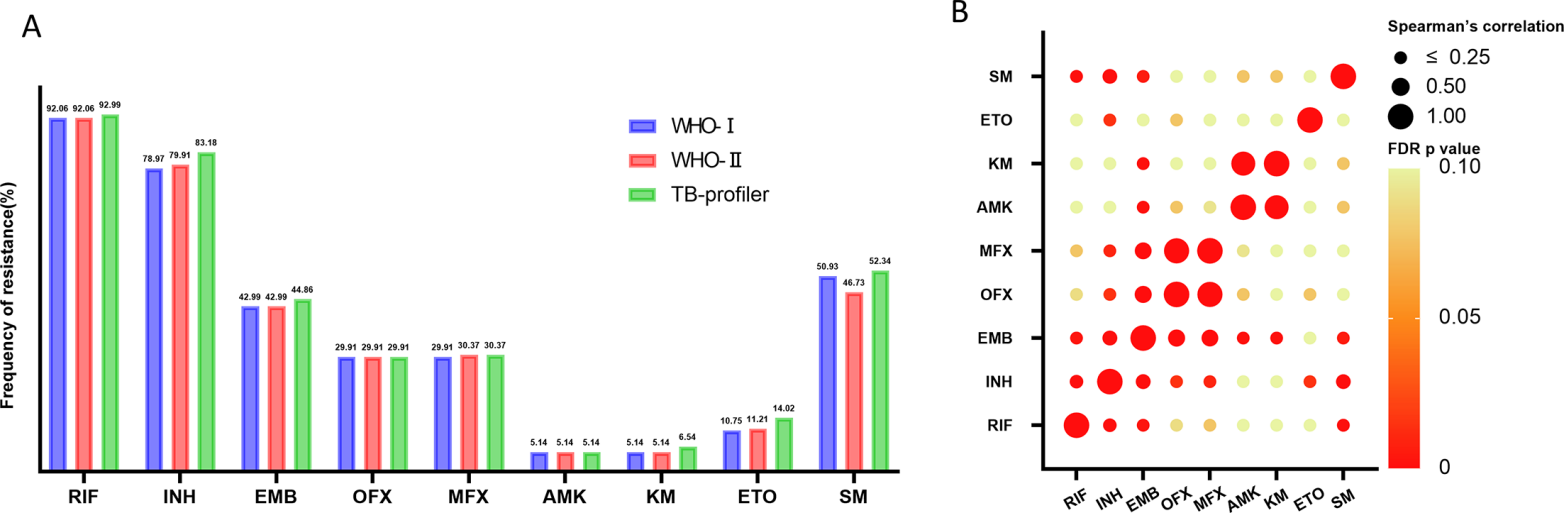
Drug resistance and correlation to nine anti-tuberculosis drugs. (**A**) The resistance rates of nine anti-tuberculosis drugs in WHO-I, WHO-II, and TB-Profiler; (**B**) a bubble plot showing Spearman’s correlation between different drugs and FDR *P* values. The magnitude of the Spearman correlation coefficient (indicated by the size of the dots) and the FDR *P* value (indicated by different colors) are indicated, where the Spearman correlation coefficients, with larger dots indicating higher correlation coefficients, are of three grades: ≤0.25, 0.50, and 1.00, respectively; and the FDR *P* values, which are graded from red to yellow, with red indicating that the *P* value is close to 0, and yellow indicating that the *P* value is close to 0.10.

According to the resistance mutations catalog by the WHO, first edition, second edition, and freely available tools (TB-Profiler) to make resistance predictions, Fig. 2A shows the frequency of resistance of 214 RR-TB strains to 9 anti-TB drugs. The resistance ratio of RIF by the three methods was 92.06% (197/214), 92.06% (197/214), and 92.99% (199/214), respectively. A total of 169 (78.97%), 171 (79.91%), and 178 (83.18%) were resistant to INH. Injectable anti-tuberculosis drugs, KM and AMK, had the lowest resistance ratio with 5.14% (11/214) according to both the WHO first and second editions, while the resistant ratio of KM and AMK by TB-Profiler was 6.54% (14/214) and 5.14% (11/214), respectively (Fig. 2A).

The relevance of resistance to nine anti-tuberculosis drugs was evaluated through bubble charts (Fig. 2B). Except for correlations between drugs in the same class, there was the highest correlation between EMB and fluoroquinolones (FQs), i.e., the correlation between EMB and OFX (Spearman’s correlation ρ = 0.446), EMB and MFX (Spearman’s correlation ρ = 0.431). To adjust for the confounding effect of the predominant lineage 2.2.1, a logistic regression analysis was performed, confirming EMB resistance as an independent predictor of FQ resistance (OR = 8.487, 95% CI: 4.138–17.405, *P* < 0.001; see Table S1 at https://doi.org/10.6084/m9.figshare.31369450). This indicates a possible link between their resistance patterns.

**Fig 3:**
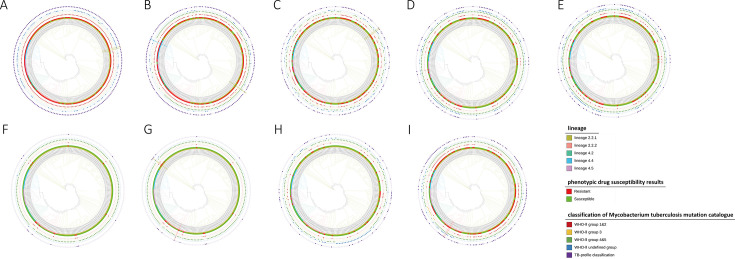
Distribution and evolution of mutations of different groups by the WHO mutation catalog for each drug. (**A**) RIF; (**B**) INH; (**C**) EMB; (**D**) OFX; (**E**) MFX; (**F**) AMK; (**G**) KM; (**H**) ETO; (**I**) SM. In the inner layer, typing evolution; in the middle layer, the results of phenotypic drug susceptibility; red solid squares: drug-resistant; green solid squares: drug-susceptible; in the second outer layer, the distribution of the six groups of mutations by WHO-II classification, solid squares indicate the presence of this mutation type, hollow squares denote the absence of this mutation type; in the outermost layer: resistance prediction by TB-Profiler. Solid purple squares represent drug-resistant strains. Hollow purple squares indicate drug-sensitive strains. Drug-resistant strains with internal group 6 mutation types are highlighted in the color of each strain’s phylogenetic lineage.

To better understand whether RR-TB strains isolated in Wenzhou exhibit lineage-specific MIC patterns, evolutionary analysis was performed on the included strains. L2 (Beijing genotype) and L4 (Euro-American genotype) lineages were observed in the 214 RR-TB strains isolated in Wenzhou. The L2 lineage demonstrated overwhelming predominance in Wenzhou, constituting 79.91% (171/214) of the isolates, whereas the L4 lineage represented the remaining 20.09% (43/214). Within the L2 lineage, the L2.2.1 subtype predominated, comprising 70.56% (151/214) of all strains, followed by L2.2.2 at 9.35% (20/214). Within the L4 lineage, the L4.4.2 subtype was the most prevalent, comprising 7.48% (16/214) of all strains, followed by L4.2.2 at 6.54% (14/214) and L4.5 at 6.07% (13/214) (Fig. 3). According to the mutation catalog by the WHO second edition, the drug-resistant mutations were categorized into five groups. For the resistance mutations not in the current WHO catalog, which were detected more than once, we tentatively defined them into internal group 6.

For RIF, 199 out of 214 MTBC isolates were determined to be resistant by pDST, 98.99% (197/199) isolates owned group 1 and/or group 2 mutations. Furthermore, newly discovered double mutations highlighted in light green (*rpoB*_1287_1295del and *rpoC*_V483G) were detected in two RIF high-level resistant isolates belonging to lineage L2.2.1 without group 1 or group 2 (Table 1; Fig. 3A).

**TABLE 1:** A list of internal group 6 mutations found in RR-TB strains^*a*^

Drug	Types of mutated genes	MIC (μg/mL)	Number of mutations	Prediction by TB-Profiler	Mutation frequency (%)	Lineage
Rifampicin	*rpoB*_1287_1295del	8 and 16	2	Resistant	100.0	2.2.1
Isoniazid	*ahpC*_−52C>T^*b*^	>4	2	Resistant	100.0 and 69.2	2.2.1 and 4.4.2
Ethambutol	*embA*_−11C>A^*b*^	4 and 8	2	Resistant	100.0 and 99.5	2.2.1
Kanamycin	*eis*_−10G>A^*b*^	10 and 20	2	Resistant	100	2.2.1 and 2.2.2
Streptomycin	*gid_*delG	4 and >32	2	Resistant	100	2.2.2

^*a*^ The internal group 6 means unclassified mutations specifically detected in drug-resistant isolates (n ≥ 2) without group 1 or group 2 by the WHO second edition mutations.

^*b*^ Candidate resistance genes that appear in the WHO second edition mutation catalog in *Mycobacterium tuberculosis* complex.

For INH, mutations catalog (group 1 and/or group 2) were associated with 95.00% (171/180) of phenotypic resistance (Fig. 3B). Two isolates with MIC = 0.25 µg/mL and >4 µg/mL harbored double mutations *katG*_Q127P and *katG*_N138H, both of which were categorized into group 3. Notably, mutations in the promoter *ahpC*_−52C>T (internal group 6) were observed in two INH-resistant isolates with MIC >4 µg/mL, one belonged to L2.2.1 and the other to L4.4.2 (Table 1). Among 102 EMB-resistant isolates, 90.20% (92/102) were associated with mutations in group 1 and/or group 2 (Fig. 3C). Mutations in internal group 6 were detected in two EMB-resistant isolates belonging to L2.2.1, both of which harbored *embA*_−11C>A with MICs of 4 and 8 μg/mL.

Mutations in group 1 and/or group 2 accounted for 96.67% (58/60) and 93.65% (59/63) for the resistance of OFX and MFX, respectively (Fig. 3D and E). The remaining resistant isolates were observed to have individual undefined mutations, which may not be associated with FQ resistance. The resistant ratio of KM and AMK was relatively lower, i.e., 5.14% (11/214) for AMK and 6.54% (14/214) for KM (Fig. 3F and G). In addition, all the AMK resistance was associated with mutations in group 1 and/or group 2. Among three KM-resistant isolates without group 1 or group 2 mutations, mutation *eis*_−10G>A was observed in two isolates (Table 1). Mutations in group 1 or group 2 accounted for 72.73% (24/33) of ETO resistance. Eight isolates (24.24%) were observed with undefined individual mutations, which suggested the presence of other drug-resistant mechanisms like efflux pumps (Fig. 3H). Among 113 SM-resistant isolates, 88.50% (100/113) were associated with mutations in group 1 and/or group 2 (Fig. 3I). Mutations in group 3 (*n* = 7) and group 4 or group 5 (*n* = 4) were also detected in SM-resistant isolates. Additionally, *gid*_delG (internal group 6) was observed in two SM-resistant isolates with MIC >4 µg/mL. Table 1 shows the potential drug-resistant mutations.

According to the WHO, first, second mutation catalogs, and TB-Profiler, 159, 159, and 213 resistance-associated mutations were detected, from which the top mutated genes with higher MIC values were selected to assess the level of resistance for each drug’s resistance mutations (Table 2). Among 25 *rpoB* mutations associated with RIF resistance, seven mutations (Asp435Val, His445Asp, His445Tyr, Ser450Leu, His445Arg, Gln432Leu, Asp435Gly) were associated with higher MIC values (median >16 µg/mL). For INH, the *katG* mutation (Ser315Thr and Ser315Asn) has a significantly higher range of MIC values than the *inhA* mutation (Ser94Ala). Multiple mutations in the *embB* gene loci were significantly associated with resistance to EMB, especially *embB*_Q497R associated with high-level EMB resistance. For FQs, multiple mutation sites in the *gyrA* gene (Asp94Tyr, Asp94Asn, and Asp94Gly) were significantly associated with high-level resistance.

**TABLE 2:** Levels of resistance to mutations cataloged by WHO^*a*^

Drug or mutation	Minimum inhibitory concentrations (μg/mL)	*P* value
Rifampicin (cutoff 0.5 μg/mL)		0.003
*rpoB*_D435V (Asp435Val)	>16.0 (16.0–16.0)^*b*^	
*rpoB*_H445D (His445Asp)	>16.0 (16.0–16.0)^*b*^	
*rpoB*_H445Y (His445Tyr)	>16.0 (16.0–16.0)^*b*^	
*rpoB*_S450L (Ser450Leu)	>16.0 (16.0–16.0)^*b*^	
*rpoB*_H445R (His445Arg)	>16.0 (16.0–16.0)^*b*^	
*rpoB*_Q432L (Gln432Leu)	>16.0 (16.0–16.0)^*b*^	
*rpoB*_D435G (Asp435Gly)	>16.0 (4.0–16.0)	
*rpoB*_H445Q (His445Gln)	16.0 (8.0–16.0)	
*rpoB*_D435A (Asp435Ala)	12.0 (8.0–16.0)	
*rpoB*_H445G (His445Gly)	8.0 (8.0–8.0)	
Isoniazid (cutoff 0.12 μg/mL)		0.102
*katG*_S315N (Ser315Asn)	2.0 (0.5–4.0)^*b*^	
*katG*_S315T (Ser315Thr)	2.0 (0.25–4.0)^*b*^	
*inhA*_S94A (Ser94Ala)	1.0 (0.5–1.0)	
Ethambutol (cutoff 2 μg/mL)		0.011
*embB*_Q497R (Gln497Arg)	12.0 (8.0–16.0)^*b*^	
*embA*_−12C>T	10.0 (4.0–16.0)	
*embB*_G406A (Gly406Ala)	8.0 (4.0–8.0)^*b*^	
*embB*_M306I (Met306Ile)	8.0 (4.0–8.0)^*b*^	
*embB*_M306V (Met306Val)	8.0 (4.0–16.0)	
*embB*_G406C (Gly406Cys)	6.0 (4.0–8.0)	
*embB*_M306L (Met306Leu)	4.0 (4.0–16.0)	
*embB*_D354A (Asp354Ala)	4.0 (4.0–16.0)	
Ofloxacin (cutoff 2 μg/mL)		0.024
*gyrA*_D94Y (Asp94Tyr)	16.0 (16.0–32.0)^*b*^	
*gyrA*_D94N (Asp94Asn)	16.0 (16.0–32.0)^*b*^	
*gyrA*_D94G (Asp94Gly)	16.0 (8.0–32.0)^*b*^	
*gyrA*_A90V (Ala90Val)	8.0 (4.0–32.0)^*b*^	
*gyrA*_S91P (Ser91Pro)	8.0 (8.0–16.0)^*b*^	
*gyrA*_D94A (Asp94Ala)	8.0 (4.0–8.0)^*b*^	
Moxifloxacin (cutoff 0.5 μg/mL)		0.026
*gyrA*_D94N (Asp94Asn)	8.0 (4.0–8.0)^*b*^	
*gyrA*_D94G (Asp94Gly)	8.0 (2.0–8.0)^*b*^	
*gyrA*_D94Y (Asp94Tyr)	8.0 (8.0–8.0)^*b*^	
*gyrA*_S91P (Ser91Pro)	4.0 (4.0–8.0)^*b*^	
*gyrA*_D94A (Asp94Ala)	4.0 (1.0–4.0)^*b*^	
*gyrA*_A90V (Ala90Val)	2.0 (1.0–8.0)^*b*^	
Amikacin (cutoff 4 μg/mL)		0.317
*rrs*_1401A>G	>16.0 (16.0–16.0)^*b*^	
Kanamycin (cutoff 5 μg/mL)		0.317
*rrs*_1401A>G	>40.0 (40.0–40.0)^*b*^	
Ethionamide (cutoff 2.5 μg/mL)		0.180
*inhA*_S94A (Ser94Ala)	40.0 (10.0–40.0)^*b*^	
*fabG1*_−17G>T	10.0 (10.0–10.0)	
Streptomycin (cutoff 2 μg/mL)		0.109
*rpsL*_K43R (Lys43Arg)	>32.0 (32.0–32.0)^*b*^	
*rpsL*_K88R (Lys88Arg)	16.0 (4.0–32.0)^*b*^	
*rrs*_514A>C	8.0 (4.0–32.0)^*b*^	

^*a*^ Cutoff: reference drug concentration (susceptibility threshold); data are median (IQR), unless stated otherwise.

^*b*^ High-level drug resistance mutations.

Using pDST results as the gold standard, we evaluated the resistance prediction based on WGS mutations for all 214 strains isolated in Wenzhou by the WHO, first, second, and TB-Profiler. When stratified by the predominant lineages, the sensitivity for first-line anti-TB drugs (RIF, INH, and EMB) remained above 89% in both lineage 2 and lineage 4, while the specificity for INH and EMB exceeded 80% in both lineages. Since all included strains were RR-TB, the specificity for RIF could not be calculated (Table 3). There were 15 isolates with pDST susceptible (MIC between 0.25 and 0.5 μg/mL) harboring RIF-resistant mutations, presenting *rpoB* L430P (*n* = 8), H445N (*n* = 6), and S450W (*n* = 1). Among these, L430P and H445N were considered as borderline RIF resistance-associated mutations by the WHO second edition catalog. Notably, TB-Profiler identified a higher number of true positives for most drugs, contributing to its higher overall sensitivity compared to the WHO catalogs (Table 3). Except for SM, the false negative number of the remaining tested drugs by the WHO second edition was lower than that of the WHO first edition, which was mainly caused by the increase of resistance mutations in group 1 and group 2. For ETO, the number of false-positive isolates identified by the WHO first, second catalogs, and TB-Profiler was 10, 9, and 3, respectively. Among these, four isolates with MIC = 2.5 µg/mL (breakoff = 5 µg/mL) harbored the mutations in the promoter *fabG1*-15C>T (group 1), suggesting the need for a borderline category. In addition, we also evaluated the consistency between the WHO first, second, and TB-Profiler in predicting drug resistance. Compared to the WHO first edition, the consistency between TB-Profiler and the WHO second edition improved for all nine drugs tested. For example, the consistency for INH increased from 0.839 to 0.881 (see Table S2 at https://doi.org/10.6084/m9.figshare.31369450).

**TABLE 3:** Genetic-based predictions of resistance by the WHO mutation catalog and TB-Profiler^*h*^

MTBC lineage	Number of isolates	Drug	Table of contents	True positives^*a*^	False positives^*b*^	True negatives^*c*^	False negatives^*d*^	PPV^*e*^ (%)	NPV^*e*^ (%)	Sensitivity^*f*^ (95% CI)	Specificity^*f*^ (95% CI)
Lineage 2	171	Rifampicin	I	156	13	0	2	92.31	NA	98.73 (95.03–99.78)	NA^*g*^
			II	156	13	0	2	92.31	NA	98.73 (95.03–99.78)	NA
			TB-Profiler	158	13	0	0	92.40	NA	100.00 (97.04–100.00)	NA
		Isoniazid	I	134	1	28	8	99.26	77.78	94.37 (88.83–97.36)	96.55 (80.37–99.82)
			II	136	1	28	6	99.27	82.35	95.77 (90.63–98.27)	96.55 (80.37–99.82)
			TB-Profiler	141	2	27	1	98.60	96.43	99.30 (95.55–99.96)	93.10 (75.79–98.80)
		Ethambutol	I	75	0	87	9	100.00	90.63	89.29 (80.16–94.68)	100.00 (94.73–100.00)
			II	75	0	87	9	100.00	90.63	89.29 (80.16–94.68)	100.00 (94.73–100.00)
			TB-Profiler	79	0	87	5	100.00	94.57	94.05 (86.04–97.79)	100.00 (94.73–100.00)
		Ofloxacin	I	49	0	121	1	100.00	99.18	98.00 (87.99–99.90)	100.00 (96.17–100.00)
			II	49	1	120	1	98.00	99.17	98.00 (87.99–99.90)	99.17 (94.81–99.96)
			TB-Profiler	49	1	120	1	98.00	99.17	98.00 (87.99–99.90)	99.17 (94.81–99.96)
		Moxifloxacin	I	49	0	120	2	100.00	98.36	96.08 (85.41–99.32)	100.00 (96.13–100.00)
			II	50	0	120	1	100.00	99.17	98.04 (88.21–99.90)	100.00 (96.13–100.00)
			TB-Profiler	50	0	120	1	100.00	99.17	98.04 (88.21–99.90)	100.00 (96.13–100.00)
		Amikacin	I	11	0	160	0	100.00	100.00	100.00 (67.86–100.00)	100.00 (97.08–100.00)
			II	11	0	160	0	100.00	100.00	100.00 (67.86–100.00)	100.00 (97.08–100.00)
			TB-Profiler	11	0	160	0	100.00	100.00	100.00 (67.86–100.00)	100.00 (97.08–100.00)
		Kanamycin	I	11	0	157	3	100.00	98.13	78.57 (48.82–94.29)	100.00 (97.02–100.00)
			II	11	0	157	3	100.00	98.13	78.57 (48.82–94.29)	100.00 (97.02–100.00)
			TB-Profiler	14	0	157	0	100.00	100.00	100.00 (73.24–100.00)	100.00 (97.02–100.00)
		Ethionamide	I	19	4	138	10	82.61	93.24	65.52 (45.66–81.40)	97.18 (92.50–99.09)
			II	20	6	136	9	76.92	93.79	68.97 (49.05–84.02)	95.77 (90.63–98.27)
			TB-Profiler	26	10	132	3	72.22	97.78	89.66 (71.50–97.29)	92.96 (87.09–96.38)
		Streptomycin	I	97	5	67	2	95.10	97.10	97.98 (92.19–99.65)	93.06 (83.86–97.42)
			II	95	1	71	4	98.96	94.67	95.96 (89.39–98.70)	98.61 (91.46–99.93)
			TB-Profiler	99	5	67	0	95.19	100.00	100.00 (95.35–100.00)	93.06 (83.86–97.42)
Lineage 2.2.1	151	Rifampicin	I	136	13	0	2	91.28	NA	98.55 (94.33–99.75)	NA
			II	136	13	0	2	91.28	NA	98.55 (94.33–99.75)	NA
			TB-Profiler	138	13	0	0	91.39	NA	100.00 (96.62–100.00)	NA
		Isoniazid	I	114	1	28	8	99.13	77.78	93.44 (87.08–96.92)	96.55 (80.37–99.82)
			II	116	1	28	6	99.15	82.35	95.08 (89.15–97.99)	96.55 (80.37–99.82)
			TB-Profiler	121	2	27	1	98.37	96.43	99.18 (94.85–99.96)	93.10 (75.79–98.80)
		Ethambutol	I	65	0	79	7	100.00	91.86	90.28 (80.42–95.67)	100.00 (94.22–100.00)
			II	65	0	79	7	100.00	91.86	90.28 (80.42–95.67)	100.00 (94.22–100.00)
			TB-Profiler	69	0	79	3	100.00	96.34	95.83 (87.50–98.92)	100.00 (94.22–100.00)
		Ofloxacin	I	39	0	111	1	100.00	99.11	97.50 (85.27–99.87)	100.00 (95.83–100.00)
			II	39	1	110	1	97.50	99.10	97.50 (85.27–99.87)	99.10 (94.36–99.95)
			TB-Profiler	39	1	110	1	97.50	99.10	97.50 (85.27–99.87)	99.10 (94.36–99.95)
		Moxifloxacin	I	39	0	110	2	100.00	98.21	95.12 (82.19–99.15)	100.00 (95.79–100.00)
			II	40	0	110	1	100.00	99.10	97.56 (85.59–99.87)	100.00 (95.79–100.00)
			TB-Profiler	40	0	110	1	100.00	99.10	97.56 (85.59–99.87)	100.00 (95.79–100.00)
		Amikacin	I	7	0	144	0	100.00	99.31	100.00 (56.09–100.00)	100.00 (96.76–100.00)
			II	7	0	144	0	100.00	99.31	100.00 (56.09–100.00)	100.00 (96.76–100.00)
			TB-Profiler	7	0	144	0	100.00	99.31	100.00 (56.09–100.00)	100.00 (96.76–100.00)
		Kanamycin	I	7	0	142	2	100.00	98.61	77.78 (40.19–96.05)	100.00 (96.72–100.00)
			II	7	0	142	2	100.00	98.61	77.78 (40.19–96.05)	100.00 (96.72–100.00)
			TB-Profiler	9	0	142	0	100.00	100.00	100.00 (62.88–100.00)	100.00 (96.72–100.00)
		Ethionamide	I	17	4	121	9	80.95	93.08	65.38 (44.37–82.06)	96.80 (91.52–98.97)
			II	18	6	119	8	75.00	93.70	69.23 (48.10–84.91)	95.20 (89.40–98.03)
			TB-Profiler	23	10	115	3	69.70	97.46	88.46 (68.72–96.97)	92.00 (85.41–95.88)
		Streptomycin	I	90	3	58	0	96.77	100.00	100.00 (94.90–100.00)	95.08 (85.40–98.72)
			II	90	1	60	0	98.90	100.00	100.00 (94.90–100.00)	98.36 (90.02–99.91)
			TB-Profiler	90	3	58	0	96.77	100.00	100.00 (94.90–100.00)	95.08 (85.40–98.72)
Lineage 4	43	Rifampicin	I	41	2	0	0	95.35	NA	100.00 (89.33–100.00)	NA
			II	41	2	0	0	95.35	NA	100.00 (89.33–100.00)	NA
			TB-Profiler	41	2	0	0	95.35	NA	100.00 (89.33–100.00)	NA
		Isoniazid	I	35	0	5	3	100.00	62.50	92.11 (77.52–97.94)	100.00 (46.29–100.00)
			II	35	1	4	3	97.22	57.14	92.11 (77.52–97.94)	80.00 (29.88–98.95)
			TB-Profiler	37	1	4	1	97.37	80.00	97.37 (84.57–99.86)	80.00 (29.88–98.95)
		Ethambutol	I	17	0	25	1	100.00	96.15	94.44 (70.63–99.71)	100.00 (83.42–100.00)
			II	17	0	25	1	100.00	96.15	94.44 (70.63–99.71)	100.00 (83.42–100.00)
			TB-Profiler	17	0	25	1	100.00	96.15	94.44 (70.63–99.71)	100.00 (83.42–100.00)
		Ofloxacin	I	9	0	33	1	100.00	97.06	90.00 (54.12–99.48)	100.00 (87.02–100.00)
			II	9	0	33	1	100.00	97.06	90.00 (54.12–99.48)	100.00 (87.02–100.00)
			TB-Profiler	10	0	33	0	100.00	100.00	100.00 (65.55–100.00)	100.00 (87.02–100.00)
		Moxifloxacin	I	9	0	31	3	100.00	91.18	75.00 (42.84–93.31)	100.00 (86.27–100.00)
			II	9	0	31	3	100.00	91.18	75.00 (42.84–93.31)	100.00 (86.27–100.00)
			TB-Profiler	10	0	31	2	100.00	93.94	83.33 (50.88–97.06)	100.00 (86.27–100.00)
		Amikacin	I	0	0	43	0	NA	100.00	NA	100.00 (89.79–100.00)
			II	0	0	43	0	NA	100.00	NA	100.00 (89.79–100.00)
			TB-Profiler	0	0	43	0	NA	100.00	NA	100.00 (89.79–100.00)
		Kanamycin	I	0	0	43	0	NA	100.00	NA	100.00 (89.79–100.00)
			II	0	0	43	0	NA	100.00	NA	100.00 (89.79–100.00)
			TB-Profiler	0	0	43	0	NA	100.00	NA	100.00 (89.79–100.00)
		Ethionamide	I	4	1	38	0	80.00	100.00	100.00 (39.58–100.00)	97.44 (84.92–99.87)
			II	4	2	37	0	66.67	100.00	100.00 (39.58–100.00)	94.87 (81.37–99.11)
			TB-Profiler	4	4	35	0	50.00	100.00	100.00 (39.58–100.00)	89.74 (74.84–96.66)
		Streptomycin	I	12	1	28	2	92.31	93.33	85.71 (56.15–97.49)	96.55 (80.37–99.82)
			II	5	0	29	9	100.00	76.32	35.71 (13.98–64.37)	100.00 (85.44–100.00)
			TB-Profiler	13	2	27	1	86.67	96.43	92.86 (64.17–99.63)	93.10 (75.79–98.80)

^*a*^ True positives (TP) indicate the number of phenotypically resistant samples that are correctly identified as resistant.

^*b*^ False positives (FP) indicate the number of phenotypically susceptible samples that are falsely identified as resistant.

^*c*^ True negatives (TN) indicate the number of phenotypically susceptible samples that are correctly identified as susceptible.

^*d*^ False negatives (TN) indicate the number of phenotypically resistant samples that are incorrectly identified as susceptible.

^*e*^ PPV, positive predictive value; NPV, negative predictive value.

^*f*^ Sensitivity = TP/(TP + FN) × 100%; specificity = TN/(TN + FP) × 100%.

^*g*^ NA, not applicable.

^*h*^ As all 214 included strains were rifampicin-resistant, specificity and NPV for rifampicin could not be calculated. Ⅰ: WHO first edition of mutations catalogue in *Mycobacterium tuberculosis* complex. Ⅱ: WHO second edition of mutations catalogue in *Mycobacterium tuberculosis* complex.

## DISCUSSION

Compared with pDST by MYCOTB plates, we have shown that the second edition WHO mutations catalog substantially improves the diagnosis of drug resistance. Resistance prediction based on WGS mutations is also more suitable for borderline resistance-associated mutations than pDST (15). With the expansion of the MTBC WGS database, especially including more infrequent mutations restricted to geographical areas, more mutations associated with drug resistance would be covered.

Previous studies have shown that the first catalog mutation improves the detection of drug resistance of MTBC with specificities more than 99.6% for the four first-line anti-tuberculosis drugs, while the sensitivity was relatively lower (85.4% for INH and 73.4% for RIF), which may be attributed to the limited number of resistant cases according to pDST (16). Our study focused on a well-characterized RR-TB cohort to assess the updated second edition catalog, particularly within the locally dominant Beijing lineage (lineage 2, *n* = 171). To provide a nuanced evaluation of its performance and address potential spectrum bias inherent in our RR-TB-focused design, we stratified predictive metrics by the predominant MTBC lineages (Table 3). This analysis revealed markedly improved sensitivities within the key Beijing lineage, with values reaching 98.73% for RIF, 95.77% for INH, and 89.29% for EMB, demonstrating the catalog’s enhanced utility for epidemic strains. Performance remained strong in the Euro-American lineage (L4), with sensitivities of 100.00%, 92.11%, and 94.44% for RIF, INH, and EMB, respectively. Additionally, our previous study also demonstrated that a borderline RIF resistance-associated mutation was one of the main causes for RIF susceptibility by MGIT960 and resistance by Gene Xpert (17). Thus, the updated catalog demonstrated robust and consistently high performance across lineages, not only showing improved sensitivity over its predecessor but also surpassing pDST, particularly in detecting critical borderline resistance, thereby providing a refined and generalizable tool for RR-TB management.

In the analysis of drug resistance correlations, we found that, in addition to strong associations between drugs of the same class (e.g., OFX and MFX, AMK and KM), EMB and FQs also showed a notable correlation. Given the widespread use and easy accessibility of FQs in the treatment of common bacterial infections, their resistance rate among MDR-TB patients in China has risen markedly. As EMB is an essential component of TB regimens, EMB-resistant patients may demonstrate concurrent FQ resistance (18, 19). Epidemiological evidence confirms this EMB-FQ association, with multivariable logistic regression analysis identifying EMB resistance as a risk factor for FQ resistance (18), consistent with our findings.

Rare variants are frequently associated with specific lineages and restricted to geographical areas or arise very rarely. For this reason, they are either poorly represented or even not included in most diagnostic mutation catalogs. Our study found some rare mutations, which may be potential resistance-associated mutations, i.e., *rpoB* indel mutations for RIF, *ahpC*_−52C>T for INH, *embA*_−11C>A for EMB, and *eis*_−10G>A for KM. Notably, all three TB drug resistance analysis tools (TB-Profiler, Mykrobe, and SAM-TB) reported *eis*_−10G>A and *embA*_−11C>A as resistance-conferring mutations. In contrast, the *ahpC*_−52C>T mutation was only reported by TB-Profiler, as it is not covered in the mutation catalogs of Mykrobe or SAM-TB. These mutations were observed among isolates of the predominant Beijing lineage 2.2.1. (Table 1). Previous studies have reported that lineage L2.2.1 can exhibit lineage-specific resistance patterns (20, 21). In our cohort, however, lineage 2.2.1 was highly prevalent overall, suggesting that the observed co-occurrence may reflect this background distribution. The potential role of these mutations in conferring drug resistance warrants further investigation in larger, lineage-balanced studies. For RIF, the mutations in *rpoC* were defined to be not associated with resistance according to the WHO second edition catalog (9). However, two RIF-resistant isolates belonging to lineage 2.2.1 harbored *rpoB*_1287_1295del and *rpoC*_V483G double mutation. Although *rpoC*_V483G is listed in WHO-II as “not associated with resistance,” it has repeatedly been described as a compensatory mutation that restores the fitness cost imposed by rpoB mutations without increasing rifampicin MICs (22, 23). In our cohort, both rifampicin-resistant isolates harboring the *rpoB*_1287_1295del (9-bp in-frame deletion) mutation co-occurred with *rpoC*_V483G. Notably, these two isolates belong to the L2.2.1 sub-lineage, which is predominant locally. This specific double mutation was absent in the remaining 197 rifampicin-resistant isolates, suggesting it is a lineage-restricted co-occurrence rather than a general resistance mechanism. We therefore emphasize that such compensatory changes should be interpreted with caution. While they may aid in phylogenetic tracking, they are not recommended for clinical resistance prediction. Whether this combination represents a synergistic resistance mechanism or merely a local phylogenetic marker requires functional validation. Until then, it should not inform adjustments to the WHO mutation grading system.

For INH, we identified one infrequent resistance-associated mutation in *ahpC_*−52C>T in two MDR isolates with MIC >4 µg/mL (one of which belonged to lineage 2.2.1), which is predicted to be susceptible by the second edition of the resistance mutations catalog. Furthermore, all the INH-susceptible strains in our study did not own this promoter mutation *ahpC*_−52C>T. Similarly, Yu et al. demonstrated that a single gene mutation in *ahpC*_−52C>T was detected in INH-resistant isolates from China with MIC above 4 μg/mL (24). In addition, Kulkarni et al. identified *ahpC*_−52C>T as one of the six compensatory mutations associated with isoniazid resistance through regression analysis. Although *ahpC* mutations do not directly cause resistance, they can enhance the sensitivity of predicting INH resistance (25).

Previous studies showed the resistance prediction based on WGS mutations for EMB is poor with the sensitivity of 50%–78.6% (15, 26). In our study, sensitivities for EMB exceeded 89% in both lineage 2 and lineage 4, which hints that new resistance-associated genes need to be explored. In our study, two EMB-resistant isolates belonging to lineage 2.2.1 harbored the *embA*_−11C>A mutation, which was consistent with what was previously reported that *embA*_−11C>A mutations were found in the EMB-resistant isolates in China with absence of mutations in *embB* (27, 28). Further evaluation of this mutation with EMB resistance needs to be explored.

Mutations in the promoter region of *eis* (Rv2416c) were considered as the mechanism of resistance to KM. Furthermore, *eis*-10G>A was considered as resistance to KM/AMK by Cepheid Xpert MTB/XDR and Hain GenoType MTBDRsl; previous studies showed that *eis*-10G>A was associated with low level of KM resistance, not AMK resistance (29, 30). In our study, two MTBC isolates (one belonged to lineage 2.2.1) were determined to be KM-resistant (MIC = 10 and 20 μg/mL), while they were still susceptible to AMK. According to the second edition mutation catalog, *eis*-10G>A was considered as not associated with drug resistance (group 5). In addition, TB-Profiler also defined *eis*-10G>A as resistant mutations, which also suggested that this mutation was highly related to drug resistance. We anticipate that when the samples in the WHO catalog grow, more resistance mutations will be covered.

There are some limitations in our study. First, our cohort was restricted to rifampicin-resistant, culture-positive isolates from a single city. This design not only introduces spectrum bias and limits lineage diversity, resulting in lineage skew, but also reduces statistical power and compromises the generalizability of our findings. Therefore, validation in larger, geographically diverse cohorts is required before national implementation can be considered. Our study is further limited by the fact that only a single isolate from each included patient underwent WGS analysis. Second, borderline RIF resistance mutations (e.g., *rpoB* L430P, H445N) detected by Xpert but often phenotypically susceptible may also contribute to the observed low specificity of WGS-based prediction for RIF. Notably, the clustering of MIC values (0.25–0.5 μg/mL) for most such borderline isolates around the current critical concentration (0.5 μg/mL) further suggests that the established phenotypic breakpoint may not optimally distinguish isolates harboring these specific borderline mutations, highlighting a potential future need for refined interpretive criteria. Third, the use of the commercially available MYCOTB plate for pDST resulted in a fixed drug panel that included WHO-obsolete agents (e.g., ofloxacin) and necessitated the use of manufacturer-specified, non-standardized breakpoints for several drugs. Fourth, the pDST of first-line drug pyrazinamide was performed in acidic medium, making it not be successfully inoculated onto microtiter plates (such as MYCOTB). Thus, pyrazinamide was not included in the drug resistance prediction based on WGS mutations. Future studies could improve completeness by incorporating validated alternative methods, such as the BACTEC MGIT PZA kit. Additionally, RIF susceptibility was determined using the MYCOTB plate method in our study. Results from other pDST systems, such as MGIT960, require further validation. Furthermore, the depth-dependent sensitivity of our variant-calling pipeline (5% allele frequency) may have missed ultra-low-frequency *katG* mutations that could explain INH resistance in the two *ahpC*_−52C>T isolates; deeper sequencing or targeted *katG* amplicon analysis would be required to rule out emerging hetero-resistance. Importantly, the identified potential resistance-associated mutations have not been functionally validated (e.g., through isogenic editing), and thus their association with resistance remains correlative. Finally, the high proportion of the Beijing lineage 2.2.1 and the resulting skewed lineage distribution in our cohort limit the interpretation of potential lineage-specific mutation patterns and increase the likelihood of chance co-occurrence.

In conclusion, our study showed that resistance prediction by catalogs of mutations was overall excellent in Wenzhou, China. Resistance prediction based on WGS mutations offered promising avenues for the tailoring of drug-resistant tuberculosis treatment, especially for diagnosing the borderline drug resistance. We also identified mutations not in the catalog, relevant at the local level, which might give insights for the future update of the catalog. With the generation of more data originating from different geographic areas, newly identified mutations would substantially improve the performance of the resistance mutation catalog.

## Data Availability

The raw whole-genome sequencing data generated and analyzed during this study have been deposited in the GSA database of the China National Center for Bioinformation, under BioProject accession number PRJCA054023. The data set is publicly accessible at the persistent link https://ngdc.cncb.ac.cn/gsa/browse/CRA036351. Additionally, the supplemental tables are available at https://doi.org/10.6084/m9.figshare.31369450. All data relevant to this study are available from the corresponding author upon reasonable request.
